# Symmetry-constrained hybrid quantum-classical convolutional neural networks for rotation-robust face recognition

**DOI:** 10.3389/frai.2026.1878911

**Published:** 2026-07-02

**Authors:** S. Sony Priya, R. I. Minu

**Affiliations:** Department of Computing Technologies, School of Computing, SRM Institute of Science and Technology, Kattankulathur, Tamil Nadu, India

**Keywords:** equivariant quantum circuits, face recognition, geometric quantum machine learning, hybrid quantum–classical networks, Klein four-group, quantum machine learning

## Abstract

Face recognition systems struggle when faces appear at different orientations. Standard convolutional neural networks handle translation well but have no built-in way to deal with rotations or reflections. Quantum neural networks offer a different kind of expressiveness, but most existing designs ignore spatial symmetry altogether. This paper introduces Eq-MG-QCNN, a hybrid quantum–classical model that builds rotation symmetry directly into the quantum circuit. The quantum filter is designed to be exactly equivariant under the Klein four-group, which covers horizontal flips, vertical flips, and 180° rotations. This is done through two mechanisms: sharing rotation parameters across all qubits (as required by orbit analysis), and connecting all qubit pairs with symmetric CZ gates (forming a complete K₄ graph). The model is tested on the ORL and Yale face databases under four rotation angles (0°, 90°, 180°, 270°) and compared against a classical CNN, a classical equivariant CNN, and the MG-QCNN quantum baseline. All experiments use noiseless quantum simulation. Eq-MG-QCNN reaches 94.3% best accuracy on ORL and 89.9% on Yale with only six quantum parameters, two fewer than the baseline. The model also shows low rotational variation across all four test angles. These results suggest that embedding group symmetry into quantum circuits is a practical way to build orientation-stable feature extractors for face recognition.

## 1 Introduction

Face recognition underpins a wide range of applications including biometric authentication, security surveillance, and human–computer interaction (Laishram et al., 2024). A standard recognition pipeline consists of three stages: face detection, feature extraction, and classification (Adjabi et al., 2020). Because the datasets used in this work contain pre-cropped and aligned facial images, we focus on the second and third stages only.

Convolutional neural networks (CNNs) have become the dominant tool for these stages, owing to their ability to learn spatial features directly from pixels (Goodfellow I et al., 2016; Alzubaidi et al., 2021). Through a hierarchy of convolution and pooling operations, CNNs progressively build more abstract representations—from edges and textures to complete face parts and identities (Gu et al., 2018). However, as datasets and models grow, CNNs face increasing challenges in computational cost and energy consumption (Hur et al., 2022).

Quantum machine learning (QML) offers an alternative paradigm by exploiting superposition, entanglement, and quantum parallelism to perform computations in an exponentially large Hilbert space (Nielson et al., 2010). Quantum convolutional neural networks (QCNNs) were introduced in Cong et al. (2019) and have since demonstrated promise for image processing and pattern recognition with logarithmic circuit depth. However, fully quantum models remain infeasible on current hardware because of gate-level noise, short coherence times, and limited qubit counts in the noisy intermediate-scale quantum (NISQ) era (Ranga et al., 2024). A further difficulty is the barren plateau phenomenon, in which the variance of the cost gradient vanishes exponentially with the number of qubits, rendering training of deep variational circuits intractable (McClean et al., 2018; Qi et al., 2023; Cunningham and Zhuang, 2024; Gelman, 2024).

To address these hardware limitations, recent work has adopted hybrid designs that combine classical deep learning with shallow parameterised quantum circuits (PQCs) (Liu et al., 2021; Hur et al., 2022; Wang et al., 2024). In a typical hybrid model, the quantum layer performs feature extraction while the classical network performs downstream classification, providing a practical middle ground between pure classical and pure quantum approaches.

A central shortcoming of most variational quantum models, including many hybrid ones, is the absence of geometric inductive priors. Faces appear in the real world under arbitrary in-plane rotations, reflections, and occlusions. Classical CNNs handle translation via weight sharing, but standard variational quantum circuits make no such provision for spatial symmetry. Geometric quantum machine learning (GQML) (Larocca et al., 2022; Ragone et al., 2023) addresses this gap by designing quantum circuits that are structurally equivariant to the action of a relevant symmetry group. Embedding such priors is known to improve sample efficiency, reduce parameter count, and suppress barren plateaus.

This paper makes the following contributions:

- A hybrid quantum–classical convolutional architecture in which the quantum filter is provably equivariant under the Klein four-group ℤ₂ × ℤ₂, obtained by combining orbit-constrained parameter sharing with a complete-graph K₄ entangler built from symmetric CZ gates.
- Patch-level numerical verification of exact equivariance to machine precision (*ε* ≤ 10^−15^), together with a reusable diagnostic algorithm applicable to any quantum convolutional filter.
- An empirical evaluation on the ORL and Yale face databases against classical CNN, classical equivariant CNN, and standard MG-QCNN baselines under identical training conditions, reporting per-rotation accuracy, rotational consistency, parameter counts, and training time.

The remainder of this paper is organised as follows. Section 2 reviews related work. Section 3 presents the group-theoretic foundations. Section 4 describes the data preparation and preprocessing pipeline. Section 5 details the proposed circuit architecture and the classical head. Section 6 describes the training procedure. Section 7 introduces the evaluation metrics. Section 8 presents experimental results on ORL and Yale. Section 9 discusses limitations. Section 10 concludes.

## 2 Related work

Quantum machine learning has emerged as a promising paradigm for augmenting conventional learning algorithms with quantum mechanical principles. Quantum circuit learning, introduced by Mitarai et al. (2018), established the foundation for hybrid quantum–classical learning by training PQCs with classical optimisers on NISQ-compatible hardware. Grant et al. (2018) proposed hierarchical quantum classifiers in which data re-uploading is used to increase expressive power while keeping the circuit shallow. Cong et al. (2019) subsequently introduced the QCNN, a symmetry-aware hierarchical quantum architecture inspired by classical CNNs that uses quantum-native convolution and pooling operations. Beer et al. (2020) proposed deep quantum neural networks based on quantum perceptrons, demonstrating strong generalisation and resilience to barren plateaus through end-to-end quantum training.

Hybrid quantum–classical architectures have been proposed to address the scaling and hardware limitations of fully quantum models (Liu et al., 2021; Mengoni et al., 2021; Mishra and Tsai, 2023; Çavşi Zaim et al., 2024; Wang et al., 2024; Zhu et al., 2024; Bali et al., 2025). In such designs, quantum components perform feature extraction and encoding while classical networks handle classification or regression. By confining quantum processing to shallow, expressive subroutines, hybrid models reduce demands on quantum resources and mitigate issues such as decoherence and barren plateaus (Pesah et al., 2021; Qi et al., 2023; Cunningham and Zhuang, 2024; Gelman, 2024; Alavia et al., 2025). In the face-recognition domain, Zhu et al. (2024) proposed the multi-gate QCNN (MG-QCNN), which uses independent per-qubit rotations with CNOT-ring entanglement. We adopt MG-QCNN as the primary quantum baseline in this work.

For structured image data, standard QCNNs exhibit limited generalisation because they lack geometric priors such as translational or rotational symmetry. Compounding this, NISQ hardware exposes deep quantum models to barren plateaus. GQML has emerged to address these shortcomings by embedding group-theoretic symmetries into quantum model design. Larocca et al. (2022) introduced group-invariant quantum machine learning, and Ragone et al. (2023) formalised symmetry embedding through representation theory. Nguyen et al. (2024) developed the theory of equivariant quantum neural networks, establishing the orbit constraint that motivates the parameter-sharing rule used in this work. Meyer et al. (2023) demonstrated practical advantages of exploiting symmetry in variational quantum machine learning, including improved trainability and generalisation (Schatzki et al., 2024) provided theoretical guarantees for permutation-equivariant quantum neural networks. Chang et al. (2023) explored approximately equivariant QNNs for p4m group symmetries in images, using amplitude encoding and aggregation to accommodate noise.

Classical equivariant networks offer a useful benchmark. Cohen and Welling (2016) introduced group-equivariant convolutional networks that generalise translation equivariance to finite groups of rotations and reflections. Weiler and Cesa (2019) extended this framework with general E(2)-equivariant steerable CNNs, providing principled architectures for rotation and reflection symmetries. We draw on this line of work for our classical equivariant baseline.

Building on these developments, the present work constructs a hybrid quantum–classical architecture whose quantum filter is structurally equivariant under the Klein four-group ℤ₂ × ℤ₂, the largest reflection-plus-180°-rotation subgroup that acts faithfully on a 2 × 2 pixel patch. Unlike approaches based on twirling (Chang et al., 2023), which aggregate outputs over group elements at inference time, the proposed circuit achieves equivariance through its internal structure alone, with no per-image averaging required.

Face analysis goes well beyond recognition. Tasks such as face anti-spoofing (Yu et al., 2022; Lin et al., 2025; Liu et al., 2024b; Ma et al., 2026), expression recognition under poor lighting (Xie et al., 2025), multimodal deception detection (Zuo et al., 2023), and image forgery detection (Lin et al., 2024) all need features that are stable under changes in pose and orientation. Recent work on audio-visual understanding (Ye et al., 2024), multimodal image fusion (Xie et al., 2024), and balanced transfer learning for cross-domain face anti-spoofing further highlights the importance of structured, robust representations across visual tasks.

## 3 Group theoretic foundations

Let X = {x^(i)^ ∈ ℝ^(H × W)}^ denote a dataset of N grayscale face images with class labels Y = {y^(i)^ ∈ {1, …, C}}. The hybrid model is a mapping given in Equation (1):


$$f_{\left(\theta ,w\right)}:{\left[0,\pi \right]}^{\left(H\times W\right)}\to \Delta^{\left(C-1\right)}$$ (1)

where θ collects the quantum gate angles and w the classical network weights, and Δ^(C − 1) is the (C − 1)-simplex of class probabilities. We require the quantum feature extractor inside f to be consistent with the spatial symmetries of 2 × 2 pixel patches under the action of the Klein four-group G = ℤ₂ × ℤ₂.

*Definition 1 (Equivariance).*

A map f: ℋ_in_ → ℋ_out_ is equivariant with respect to a group G if, for every g ∈ G and every input x, as expressed in Equation (2):


f(ρin(g)·x)=ρout(g)·f(x) (2)

where ρ_in_ and ρ_out_ are linear representations of G on the input and output Hilbert spaces. Intuitively, transforming the input before f is equivalent to transforming the output afterwards.

*Definition 2 (Invariance).*

Invariance is the special case in which ρ_out_(g) = I for all g ∈ G, i.e., f(g x) = f(x). The quantum filter in this work is designed to be equivariant; the classical classification head is learned and is not constrained to invariance, although training with augmentation encourages invariance at the output.

### 3.1 The ℤ₂ × ℤ₂ symmetry group

The Klein four-group G = ℤ₂ × ℤ₂ = {e, σ_x_, σᵧ, σ_x_σᵧ} is the smallest non-cyclic group, with every element its own inverse. Here e is the identity, σ_x_ is horizontal reflection (column swap), σᵧ is vertical reflection (row swap), and σ_x_σᵧ is their composition, which is equivalent to a 180° rotation about the patch centre (Table 1).

**Table 1:** Klein four-group multiplication table.

∘	e	σ_x_	σ_γ_	σ_x_σ_γ_
e	e	σ_x_	σ_γ_	σ_x_σ_γ_
σ_x_	σ_x_	e	σ_x_σ_γ_	σ_γ_
σ_γ_	σ_γ_	σ_x_σ_γ_	e	σ_x_
σ_x_σ_γ_	σ_x_σ_γ_	σ_γ_	σ_x_	e

#### 3.1.1 Group action on a 2 × 2 patch

Each 2 × 2 patch extracted from the 16 × 16 image is indexed by four pixels arranged as in Equation (3).


P=[[p00,p01],[p10,p11]]«(qn0,q1,q2,q3) (3)

where q₀ = p₀₀, q₁ = p₀₁, q₂ = p₁₀, q₃ = p₁₁ maps pixels to qubit indices. The four group elements act on these indices as the following qubit permutations, given in Equations (4)–(7):


e:(0,1,2,3)→(0,1,2,3) (4)

σx:(0,1,2,3)→(1,0,3,2) (5)

σy:(0,1,2,3)→(2,3,0,1) (6)

σxσy:(0,1,2,3)→(3,2,1,0) (7)

These permutations lift to unitary operators on the four-qubit Hilbert space (ℂ^2^) ⊗ ^4^ via tensor products of SWAP gates, as shown in Equations (8)–(10):


R(σx)=SWAP(0,1)⊗SWAP(2,3) (8)

R(σy)=SWAP(0,2)⊗SWAP(1,3) (9)

R(σxσy)=R(σx)·R(σy). (10)

These representations are unitary by construction and satisfy R(g₁)R(g₂) = R(g₁g₂) as required. Orbit Analysis and Its Implications for Circuit Design.

A key structural property of ℤ₂ × ℤ₂ acting on four qubit indices is that every single qubit falls in the same orbit, as given in Equation (11):


$$\begin{array}{c} \mathrm{Orb}(\mathrm{q0})=\{\mathrm{q0},\mathrm{\sigma x}(\mathrm{q0})=q₁,\mathrm{\sigma y}(\mathrm{q0})=q₂,\text{σxσy}(\mathrm{q0})=q₃\}\\ =\{\mathrm{q0},q₁,q₂,q₃\} \end{array}$$ (11)

**Figure 1:**
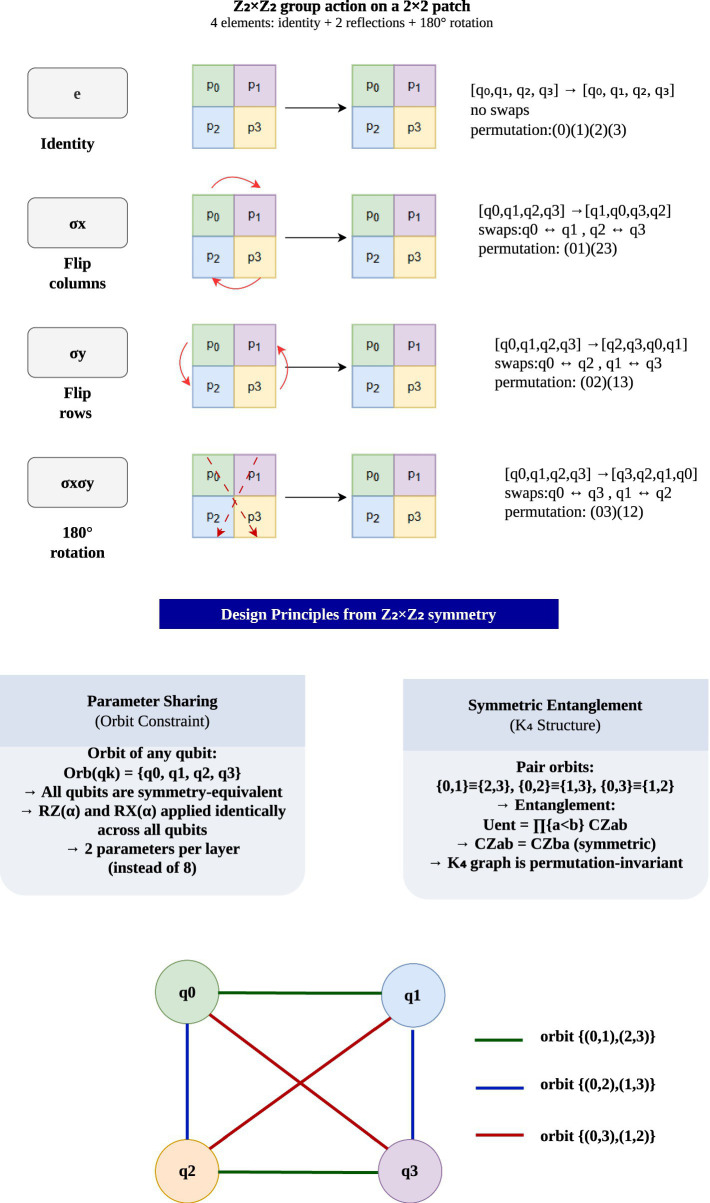
Action of the Klein four-group ℤ₂ × ℤ₂ on a 2 × 2 image patch.

Orbit transitivity—the fact that the group maps any qubit to any other—has a direct consequence for circuit design. By the orbit constraint for equivariant quantum channels (Nguyen et al., 2024), any single-qubit parameterised gate in an equivariant layer must assign the same parameter to every qubit in the orbit. If qubit q₀ carries rotation angle *α*, then qubits q₁, q₂, q₃ must all carry α, otherwise permuting the qubits under a group element would produce a different output. In practical terms, a layer that naïvely requires four independent parameters is collapsed to a single shared parameter per gate type. Across three layers with two gate types each (RZ and RX), this reduces the free parameter count from 4 × 2 × 3 = 24 to 2 × 3 = 6. These group actions and the resulting design constraints are illustrated in Figure 1.

Figure 1 shows the working of the Klein four-group ℤ₂ × ℤ₂ on a 2 × 2 image patch. The top portion presents the four group elements (identity, column swap σ_x_, row swap σᵧ, and 180° rotation σ_x_σᵧ), represented as pixel permutations and in cycle notation. The middle part illustrates the design principles derived from orbit analysis—parameter sharing (orbit constraint) and symmetric K₄ entanglement. The bottom part depicts the complete graph K₄ used for CZ entanglement, with edges coloured according to pair-orbit classes under ℤ₂ × ℤ₂.

## 4 Data preparation and preprocessing

### 4.1 Datasets

Experiments are conducted on two complementary face-recognition benchmarks. The ORL face database (Samaria and Harter, 1994) comprises 400 grayscale images distributed evenly across 40 subjects (10 images per subject), acquired under varying lighting, facial expressions, and facial details such as glasses. The Yale face database (Belhumeur et al., 1996) consists of 165 grayscale images from 15 subjects, with 11 images per subject, depicting each subject under different illumination conditions. ORL presents primarily geometric variation, while Yale presents primarily photometric variation, so together they probe the model from complementary angles.

### 4.2 Preprocessing pipeline

A four-step pipeline transforms raw images into the format required by the quantum circuit.

*Step 1*: Spatial downsampling. ORL images are obtained at 64 × 64 via scikit-learn’s fetch_olivetti_faces, which rescales the original 92 × 112 images internally. Each image is resized from 64 × 64 to 16 × 16 using bilinear interpolation as implemented in PyTorch’s F.interpolate with align_corners = False. The resulting 16 × 16 image retains sufficient structural information for identity discrimination while keeping the quantum patch dimension tractable at 2 × 2, yielding 64 patches per image.

*Step 2*: Intensity normalisation to [0, *π*]. Quantum RY angle encoding maps pixel values to rotation angles on the Bloch sphere. Rotation angles must lie in [0, π] to avoid aliasing. Each pixel is rescaled as in Equation (12):


$$\tilde{x}\{\mathrm{ij}\}=(x\{\mathrm{ij}\}-\text{xmin})/(\text{xmax}-\text{xmin}+\varepsilon )\cdot \pi$$ (12)

with ε = 10^−8^ for numerical stability, and xmin, xmax computed over the full dataset tensor. An angle of 0 encodes a qubit in |0⟩ and π encodes a qubit in |1⟩, with intermediate values encoding the Bloch-sphere latitudes in between.

**Figure 2:**
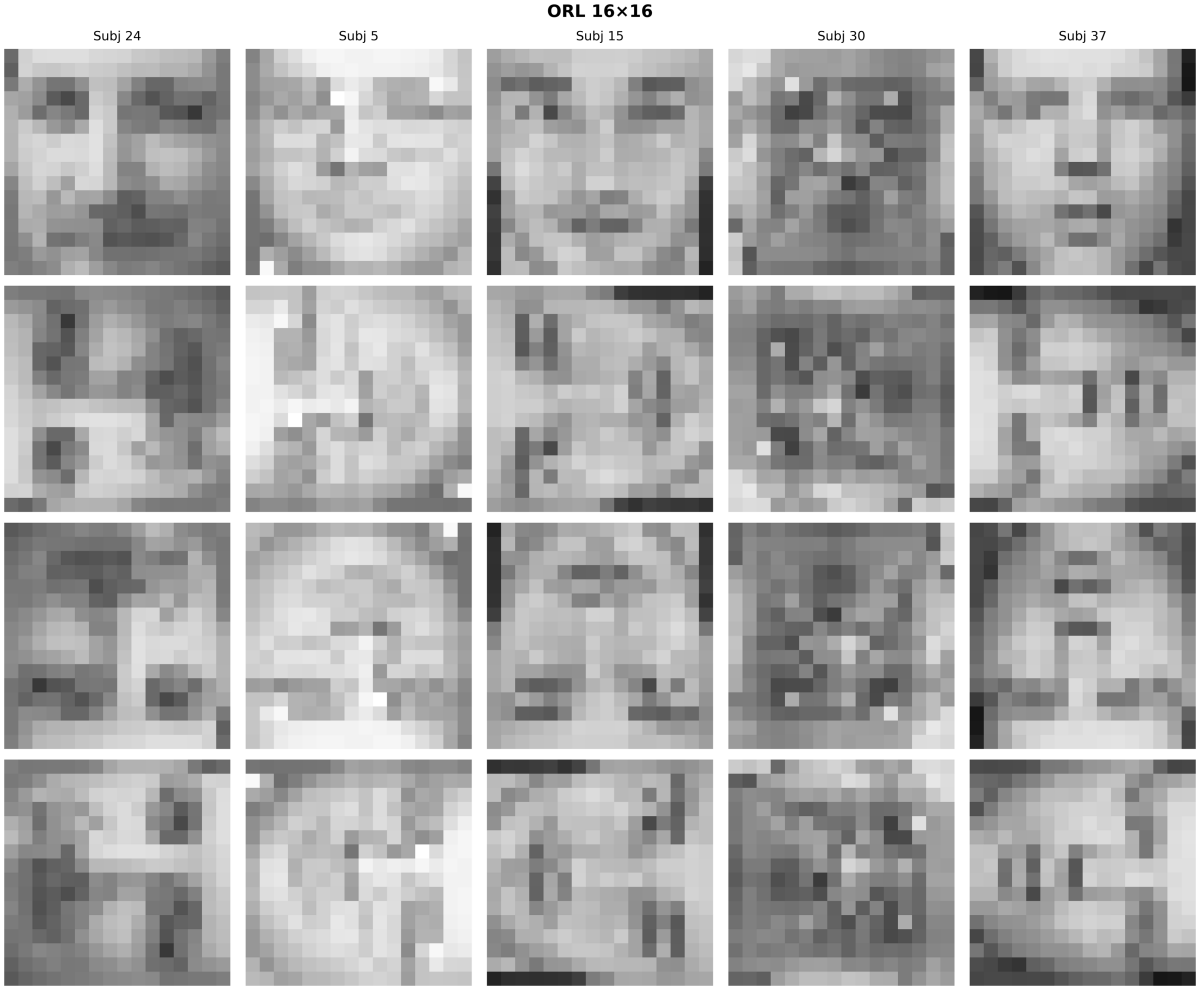
Sample ORL face images at resolution 16 × 16 after rotation augmentation.

*Step 3*: Rotational augmentation. To expose the model to a richer set of pose variations, each training image is replicated at four discrete orientations—0°, 90°, 180°, and 270°—using the torchvision functional rotate operator. This quadruples the training set: ORL yields 300 × 4 = 1,200 training images; Yale yields 123 × 4 = 492 training images after the train–test split. Class labels are invariant under rotation. Test images are evaluated separately at each orientation so that per-rotation accuracy can be measured. Sample images after this preprocessing are shown in Figure 2.

Note that the 90° and 270° rotations are not elements of ℤ₂ × ℤ₂; they belong to the cyclic group C₄. The equivariance guarantee provided by our circuit construction therefore covers the 0° (identity) and 180° cases exactly, while the 90° and 270° cases measure whether the ℤ₂ × ℤ₂-equivariance prior transfers empirically to the larger C₄ orbit. This design choice lets us separate proven equivariance from empirical rotation robustness in the evaluation. Figure 2 shows sample ORL face images at 16 × 16 resolution under four-rotation augmentation. Each column shows a single subject; each row shows the same subjects rotated by 0°, 90°, 180°, and 270° respectively.

*Step 4*: Stratified train–test split. A 75/25 split is applied before augmentation and is stratified by subject, ensuring that every subject contributes proportionally to both training and test sets. For ORL this yields 300 train / 100 test images; for Yale, 123 train / 42 test images. This avoids subject leakage and guarantees that the test set measures generalisation to unseen images of known subjects.

## 5 Quantum circuit architecture

**Figure 3:**
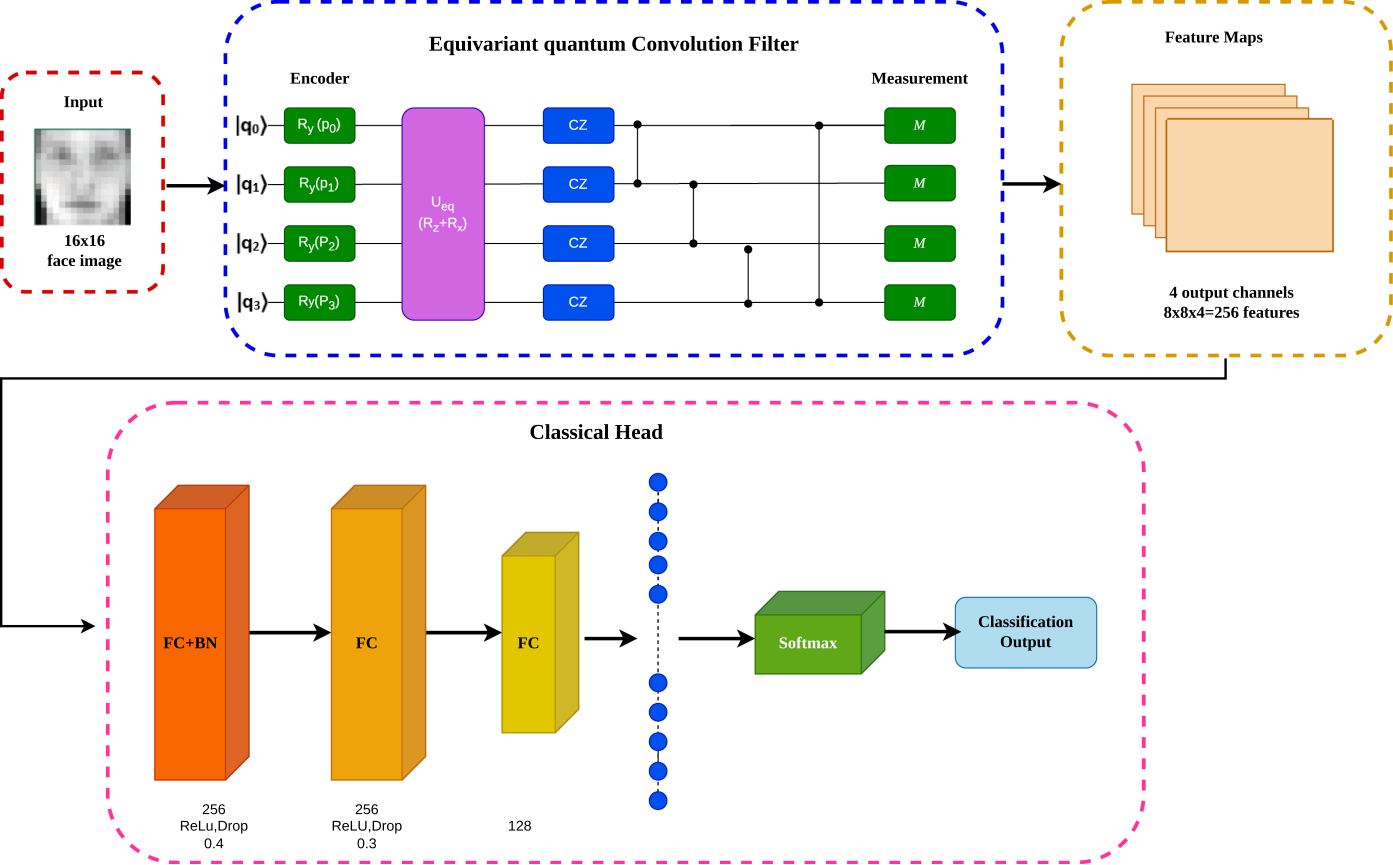
Overall workflow of proposed model.

The hybrid model processes each 16 × 16 image patch-by-patch through a four-qubit quantum circuit, then flattens the resulting feature tensor and passes it through a classical fully connected head. Figure 3 illustrates the complete workflow.

### 5.1 Angle encoding layer

The 16 × 16 image is processed with a stride-2 sliding window, producing 8 × 8 = 64 non-overlapping 2 × 2 patches. Each patch p = (p₀, p₁, p₂, p₃) ∈ [0, *π*]^4^ is encoded into a four-qubit quantum state by applying independent R_Y_ rotations, as given in Equation (13):


$$\begin{array}{c} \varepsilon (p)=⊗\{k=0\}\{3\}\;\mathrm{RY}(\mathrm{pk})\;|0\rangle \\ =⊗\{k=0\}\{3\}\;(\cos(\mathrm{pk}/2),\sin(\mathrm{pk}/2))T \end{array}$$ (13)

The resulting state |ψ_enc_⟩ is a product state; no entanglement is created during encoding. The encoding is equivariant by construction—permuting pixels under g ∈ G is equivalent to applying the corresponding qubit permutation R(g)—which follows from the independence of the R_Y_ gates across qubits.

### 5.2 MG-QCNN baseline variational circuit

The multi-gate QCNN of Zhu et al. (2024) serves as the quantum baseline. Its variational unitary applies one RZand one R_X_ gate per qubit with independent parameters, followed by a CNOT-ring entangler, as defined in Equation (14):


$$\begin{array}{c} \mathrm{UMG}(\theta )=\text{CNOTring}\cdot ⊗\{k=0\}\{3\}\;\\ \mathrm{RX}(\theta \{k+4\})\cdot ⊗\{k=0\}\{3\}\;\mathrm{RZ}(\mathrm{\theta k}) \end{array}$$ (14)

with θ = (θ₀, …, θ₇) ∈ ℝ^8^ and CNOT_ring_ = CNOT_{0 → 1}_ · CNOT_{1 → 2}_ · CNOT_{2 → 3}_ · CNOT_{3 → 0}_. The CNOT ring is asymmetric (CNOT_{a → b}_ ≠ CNOT_{b → a}_), and the cyclic ordering does not commute with R(g) for any non-identity element of ℤ₂ × ℤ₂. The circuit therefore has 8 independent parameters and no structural equivariance.

### 5.3 Eq-MG-QCNN: proposed equivariant circuit

The proposed Eq-MG-QCNN enforces exact ℤ₂ × ℤ₂ equivariance through two structural mechanisms applied simultaneously—one governing single-qubit rotations and one governing two-qubit entanglement.

*Mechanism 1: Shared parameters via orbit constraint*. Since Orb(q_k_) = {q₀, q₁, q₂, q₃} for every k, equivariance requires the same rotation angles on every qubit within each layer. The rotation block of layer ℓ is therefore, given by Equation (15):


$$\begin{array}{l} \text{Urot}(\ell )(\alpha \{2\ell \},\alpha \{2\ell +1\})\\ =⊗\{k=0\}\{3\}\;\mathrm{RX}(\alpha \{2\ell +1\})\cdot ⊗\{k=0\}\{3\}\;\mathrm{RZ}(\alpha \{2\ell \}) \end{array}$$ (15)

where α_{2ℓ}_ and α_{2ℓ + 1}_ are shared scalars — identical across all four qubits. This collapses the 8 per-layer parameters of MG-QCNN to 2 per layer, with no loss of expressivity in the equivariant subspace.

*Mechanism 2: Symmetric entanglement via K₄*. The two-qubit entangler must also commute with qubit permutations. The controlled-Z gate is intrinsically symmetric—CZ_{ab}_ = CZ_{ba}_—making it permutation-compatible. Applying CZ to all C(4,2) = 6 qubit pairs forms the complete graph K₄ on four vertices, as in Equation (16):


Uent=∏{0≤a<b≤3}CZ{ab} (16)

Since K₄ is vertex-transitive and every edge is symmetric, U_ent_ is invariant under all vertex permutations in S₄, and therefore under the subgroup ℤ₂ × ℤ₂ ⊂ S₄. The three pair orbits {(0,1), (2,3)}, {(0,2), (1,3)}, and {(0,3), (1,2)} confirm that no edge is structurally privileged.

**Figure 4:**
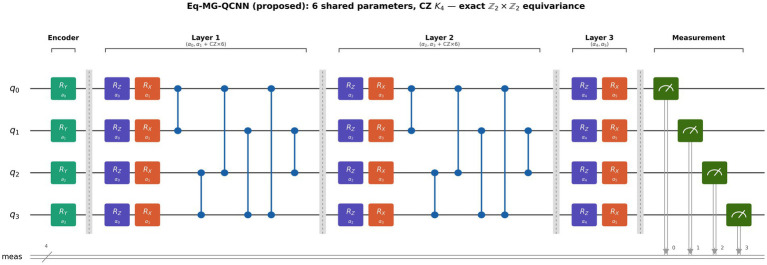
Proposed Eq-MG-QCNN circuit.

Three rotation layers are stacked with K₄ entanglement inserted between layers 1–2 and between layers 2–3, but not after layer 3 (unnecessary before measurement), as given in Equation (17):


$$\begin{array}{c} \mathrm{UEq}(\alpha )=\text{Urot}(3)(\alpha ₄,\alpha 0)\cdot \text{Uent}\cdot \text{Urot}(2)(\alpha ₂,\alpha ₃)\cdot \\ \text{Uent}\cdot \text{Urot}(1)(\alpha 0,\alpha ₁) \end{array}$$ (17)

with α = (α₀, …, α₅) ∈ ℝ^6^. The complete circuit layout is shown in Figure 4.

*Theorem 1 (Exact Equivariance of U_Eq_)*

For all g ∈ ℤ₂ × ℤ₂ and all parameter vectors α ∈ ℝ^6^, the circuit satisfies Equation (18):


$$U_{E}q(\alpha )\cdot R(g)=R(g)\cdot U_{E}q(\alpha )$$ (18)

*Proof sketch.* It suffices to show that each factor of U_Eq_ commutes with R(g). For any rotation block U_rot(ℓ)_: because all four qubits receive identical gates with the same angle, permuting qubit labels under R(g) merely reorders a product of identical operators, which leaves the total unitary unchanged. For U_ent_: the complete graph K₄ contains all C(4,2) = 6 edges, so any vertex permutation maps the edge set onto itself; combined with the symmetry of CZ, this yields R(g) U_ent_ R(g)† = U_ent_ for all g ∈ ℤ₂ × ℤ₂. Since a composition of commuting factors remains commuting, U_Eq_ inherits exact equivariance. Numerical verification over the three non-trivial group elements σ_x_, σ_γ_, σ_x_σ_γ_ for a representative parameter vector gives a maximum commutator norm below 10^−15^, confirming machine-precision equivariance.

### 5.4 Measurement and quantum feature extraction

After the variational circuit, each of the four qubits is measured in the Pauli-Z basis. The output feature vector for a given patch p is given by Equation (19):


f(p)=(Z0,Z₁,Z₂,Z₃)∈[−1,1]4 (19)

where ⟨Z_k_⟩ = ⟨ψ_out_| Z_k_ |ψ_out_⟩. Applying this filter to every 2 × 2 patch with stride 2 produces a spatial feature tensor F ∈ ℝ^(8 × 8 × 4). Flattening F yields a 256-dimensional quantum feature vector z = vec(F) ∈ ℝ^256^.

#### 5.4.1 Relationship between classical and quantum equivariance

Although both classical G-CNNs (Cohen and Welling, 2016) and the proposed Eq-MG-QCNN enforce equivariance through constrained parameter sharing, the underlying mechanisms differ in important ways. In classical G-CNNs, a single learned kernel is physically rotated and reflected under each group element, and the resulting feature map is the sum of convolutions with every transformed copy. Equivariance follows from the commutativity of convolution with spatial group actions, and the number of free parameters equals the kernel size (Ravanbakhsh et al., 2017).

In the quantum setting, equivariance arises from a different algebraic condition: the parameterised unitary U(*θ*) must commute with the group representation, *ρ*(g)U(θ) = U(θ)ρ(g) for every group element g. For Z₂ × Z₂ acting on four qubits, this condition forces two constraints simultaneously. First, because the group acts transitively on the qubit set, the orbit–stabiliser theorem requires all four qubits to share the same rotation angle, reducing the parameter count from 8 to 6. Second, the six qubit pairs partition into three orbits, each of which must carry the same entangling gate; applying CZ to all six pairs (the K₄ topology) satisfies this requirement. The entanglement constraint has no classical analogue.

A further distinction concerns approximate symmetry. Classical G-CNNs are either exactly equivariant or not. In the quantum circuit, the measurement-layer parameter *ϕ* provides a mechanism for smooth relaxation: setting ϕ = 0 yields exact equivariance, while a nonzero ϕ introduces controlled symmetry breaking that can compensate for systematic hardware errors on NISQ devices (Chang et al., 2023).

### 5.5 Classical classification head

The quantum feature vector z ∈ ℝ^256^ serves as the input to a three-layer fully connected classifier. The forward pass is, given by Equation (20):


$$\overset{⌢}{y}=\text{softmax}(W₃\cdot \sigma (W₂\cdot \sigma (\mathrm{BN}(W₁\cdot z+b₁))+b₂)+b₃)$$ (20)

with W₁ ∈ ℝ^(256 × 256)^, W₂ ∈ ℝ^(128 × 256)^, W₃ ∈ ℝ^(C × 128)^, where C = 40 for ORL and C = 15 for Yale. Here σ(·) is the ReLU activation and BN denotes batch normalisation applied after the first linear transformation. The first hidden layer has width 256, the second has width 128. Dropout with drop rates 0.4 and 0.3 is applied after the first and second hidden layers respectively, providing regularisation suited to the small datasets. Batch normalisation is placed only on the first layer, where the quantum–classical interface can introduce distribution shift as the quantum parameters evolve during training.

### 5.6 Classical baselines

Two classical models serve as baselines. The classical CNN is a conventional architecture with three convolutional blocks (32, 64, 128 channels) interleaved with batch normalisation, ReLU, 2 × 2 max pooling, and 2-D dropout, followed by a fully connected head of the same structure used for the quantum models. The classical equivariant CNN, which we denote E2-CNN following [32, 33], implements ℤ₂ × ℤ₂ equivariance by concatenating four spatially transformed copies of the input—identity, horizontal flip, vertical flip, and 180° rotation—along the channel dimension before applying standard convolutions. This is the simplest realisation of group-equivariant CNNs for a discrete reflection group and provides a fair classical point of comparison. Table 2 summarises both architectures.

**Table 2:** Classical baseline architectures.

Stage	Classical CNN	E2-CNN
Input	1 × 16 × 16	4 × 16 × 16 (ID, σ_x_, σ_γ_, σ_x_σ_γ_ stacked)
Conv block 1	3 × 3 conv, 32 ch, BN, ReLU	3 × 3 conv, 32 ch, BN, ReLU
Conv block 2	3 × 3 conv, 64 ch, BN, ReLU; 2 × 2 max-pool; Dropout2d(0.25)	3 × 3 conv, 64 ch, BN, ReLU; 2 × 2 max-pool; Dropout2d(0.25)
Conv block 3	3 × 3 conv, 128 ch, BN, ReLU; 2 × 2 max-pool; Dropout2d(0.25)	3 × 3 conv, 128 ch, BN, ReLU; AdaptiveAvgPool → 4 × 4; Dropout2d(0.25)
FC head	2048 → 256 → 128 → C (Dropout 0.4/0.3, ReLU)	2048 → 256 → 128 → C (Dropout 0.4/0.3, ReLU)

## 6 Training procedure

### 6.1 Loss function

All models are trained by minimising the cross-entropy loss in Equation (21):


$$L(\theta ,W)=-(1/N)\mathrm{\Sigma i}\Sigma_{c}y_{c}^{\left(i\right)}\log{\hat{y}}_{c}^{\left(i\right)}\;$$ (21)

### 6.2 Hybrid optimisation

Quantum parameters *α* and classical weights W are updated jointly per mini-batch via the Adam optimiser, using separate learning rates for the two parameter groups, as in Equations (22) and (23):


$$\alpha \leftarrow \alpha -\eta_{q}\cdot \text{Adam}(\nabla_{\alpha }L),\eta_{q}=0.01$$ (22)

$$W\leftarrow W-\eta_{c}\cdot \text{Adam}(\nabla_{W}L),\eta_{c}=0.001$$ (23)

The quantum learning rate is set an order of magnitude higher than the classical rate because quantum parameter gradients computed by PennyLane’s backpropagation interface are typically smoother than gradients through deep fully connected layers. Gradients are computed via exact backpropagation through the statevector simulation (diff_method = ‘backprop’), not via the parameter-shift rule. We note this distinction because parameter-shift gradients would be required on real quantum hardware; the present results therefore reflect noiseless simulation only. Gradient clipping at ‖∇‖₂ ≤ 1 is applied to prevent divergence during early epochs.

### 6.3 Initialisation and execution

Quantum parameters are initialised from (0, 0.01) to remain close to the identity transformation at the start of training, a strategy known to mitigate the barren plateau regime (Pesah et al., 2021; Qi et al., 2023). Classical weights use the PyTorch default initialisation Each experiment is trained for 30 epochs with a batch size of 16 and a fixed random seed (SEED = 42). The complete set of training hyperparameters is summarised in Table 3, and the hardware and software environment is listed in Table 4.

**Table 3:** Training hyperparameters.

Hyperparameter	Value
Optimiser	Adam (separate quantum and classical groups)
Quantum learning rate	0.01
Classical learning rate	0.001
Batch size	16
Epochs	30
Loss function	Cross-entropy
Gradient clipping	max norm 1.0
Quantum parameter init	N(0, 0.01)
Classical weight init	PyTorch default (Kaiming uniform)
Dropout rates	0.4 (layer 1), 0.3 (layer 2)
Gradient method	Backpropagation (diff_method = backprop)
Image size	16 × 16, grayscale, normalised to [0, pi]
Patch size/stride	2 × 2, stride 2 (64 patches)
Train/test split	75/25 stratified (before augmentation)
Augmentation	4 rotations (0, 90, 180, 270 degrees)
Random seed	42

**Table 4:** Experimental environment.

Component	Specification
Platform	Google Colab Pro
Quantum simulation	CPU (PennyLane default.qubit, exact statevector)
Python	3.10
Deep learning framework	PyTorch 2.x
Quantum framework	PennyLane 0.38+
Other libraries	NumPy, scikit-learn, torchvision, matplotlib

## 7 Evaluation metrics

### 7.1 Per-rotation accuracy

To assess how each model generalises across the four orientations sampled by the augmentation, test accuracy is computed separately for each rotation angle *θ* ∈ {0°, 90°, 180°, 270°}, as in Equation (24):


$$\mathrm{Acc}(\theta )=(1/N_{t}\mathrm{est})\mathrm{\Sigma i}1{\hat{y}}^{\left(i\right)}(\theta )=y^{\left(i\right)}$$ (24)

An orientation-stable model should maintain consistent accuracy across all four rotations.

### 7.2 Rotational consistency

The standard deviation of per-rotation accuracies provides a scalar summary of orientation stability at the prediction level, defined in Equation (25):


$$\text{Consistency}=\mathrm{Std}(\begin{array}{l} \mathrm{Acc}(0^{{}^{\circ}}),\mathrm{Acc}(90^{{}^{\circ}}),\\ \mathrm{Acc}(180^{{}^{\circ}}),\mathrm{Acc}(270^{{}^{\circ}}) \end{array})$$ (25)

A perfectly equivariant model with respect to the test group achieves Consistency = 0. Because our proven group is ℤ₂ × ℤ₂ rather than C₄, perfect consistency is not guaranteed; the metric therefore reports residual orientation sensitivity empirically.

### 7.3 Patch-level equivariance error

At the circuit level, the equivariance error quantifies the degree to which the feature extractor truly commutes with the group. For a trained circuit U with feature map f(p), the error is defined in Equation (26):


$$\varepsilon_{e}\text{quiv}=\max_{g\in G}\max_{p}\|f(g\cdot p)-\pi_{g}\cdot f{\left(p\right)}_{\infty }\;$$ (26)

where π_g is the output permutation corresponding to group element g. Equivariance requires f(g p) = *π*_g f(p). By construction, Eq-MG-QCNN achieves *ε*_equiv_ ≈ 10^−15^ at machine precision. MG-QCNN has ε_equiv_ > 0; the specific value depends on trained parameters and is reported in Section 8.

### 7.4 Training and verification algorithms

Algorithm 1 presents the end-to-end training procedure. The nested loop structure reflects the patch-by-patch quantum processing: for each training image in each mini-batch, the 16 × 16 image is decomposed into 64 non-overlapping 2 × 2 patches, each processed independently through the quantum circuit to build the feature tensor F.

Algorithm 1: Eq-MG-QCNN end-to-end trainingInput: Training set {(xᵢ, yᵢ)}, per-rotation test sets
Output: Trained parameters α*, W*
1: Initialise α ← (0, 0.01), W ← PyTorch default
2: Augment: X_t_ rain ← ⋃_θ∈0°, 90°, 180°, 270°_ Rot(X,θ)
3: for epoch = 1 to 30 do
4:  Shuffle training indices
5:   for each mini-batch B ⊆ X_train_ do
6:    for each image x ∈ B do
7:      for each 2×2 patch p_{ij}_ do
8:        |ψ_enc_⟩ ← ⊗_k_ R_Y_(p_k_) |0⟩
9:          for ℓ = 1 to 3 do
10:      |ψ⟩ ← ⊗_k_ R_X_(α_{2ℓ+1})_ · ⊗_k_ R_Z_(α_{2ℓ}_) |ψ⟩
11:      if ℓ < 3: |ψ⟩ ← ∏_{a<b}_ CZ_{ab}_ |ψ⟩
12:        end for
13:        F_{ij}_ ← (⟨Z₀⟩, ⟨Z₁⟩, ⟨Z₂⟩, ⟨Z₃⟩)
14:        end for
15:          z ← vec(F) ∈ ℝ²⁵⁶
16:            end for
17:            ŷ ← softmax(FC(z; W))
18:        L ← CrossEntropy(ŷ, y)
19:        Compute ∇_α L_, ∇_W L_ via backpropagation
20:          Clip ‖∇‖₂ ≤ 1.0
21:        Update α via Adam(η_q_ = 0.01); update W via Adam(η_c_ = 0.001)
22:          end for
23:      Evaluate Acc(θ) for θ ∈ {0°, 90°, 180°, 270°}
24:  end for
25:  return α*, W*

Algorithm 2 details the patch-level equivariance verification procedure. It is applied post-training to quantify *ε*_equiv_ for each trained circuit. The procedure is model-agnostic and can be applied to any quantum convolutional filter, making it a reusable diagnostic tool.

Algorithm 2: Patch-level ℤ₂ × ℤ₂ equivariance verificationInput: Trained circuit U(α), test patch p = (p₀, p₁, p₂, p₃)
Output: Maximum equivariance error ε
 1: Define group actions on patch indices:
    σ_x_(p)  = (p₁, p₀, p₃, p₂)
    σ_γ_(p)  = (p₂, p₃, p₀, p₁)
    σ_x_σ_γ_(p) = (p₃, p₂, p₁, p₀)
 2: Define corresponding output permutations:
    π_{σx}_  = (1, 0, 3, 2)
    π_{σγ}_  = (2, 3, 0, 1)
    π_{σxσγ}_ = (3, 2, 1, 0)
 3:    Compute f(p) = (⟨Z₀⟩, …, ⟨Z₃⟩) via U(α)
 4:    ε ← 0
 5:    for each g ∈ {σ_x_, σ_γ_, σ_x_σ_γ_} do
 6:    Compute f(g · p) via U(α)
 7:    Compute expected output: π_g · f(p)
 8:    ε ← max(ε, ‖f(g · p) − π_g · f(p)‖_∞)
 9:    end for
10:    return ε

## 8 Experimental results

Four models—Classical CNN, E2-CNN, MG-QCNN, and Eq-MG-QCNN—are trained and evaluated under identical data splits, augmentation procedures, optimisation hyperparameters, and random seed. Quantum circuits are simulated in PennyLane’s exact-statevector mode on CPU; classical layers use GPU when available. Each model is trained for 30 epochs with batch size 16.

### 8.1 ORL face database

Table 5 presents per-rotation accuracy, mean accuracy, best accuracy observed across 30 epochs, rotational consistency, parameter counts, and training time for all four models on ORL.

**Table 5:** Orl face database—quantitative results.

Model	0°	90°	180°	270°	Mean	Best	Std(%)	95% CI	Q-params	C-params	Time(s)
Classical CNN	84	87	84	86	85.2	85.8	1.3	[60.8, 70.2]	0	655,720	109
E2-CNN	87	89	90	89	88.8	89.7	1.1	[69.4, 78.1]	0	656,584	121
MG-QCNN (Zhu et al., 2024)	87	86	84	86	85.8	85.8	1.1	[82.9, 89.6]	8	104,360	35,677
Eq-MG-QCNN	92	93	92	93	92.5	94.3	0.5	[89.6, 94.9]	6	104,360	111,144

**Figure 5:**
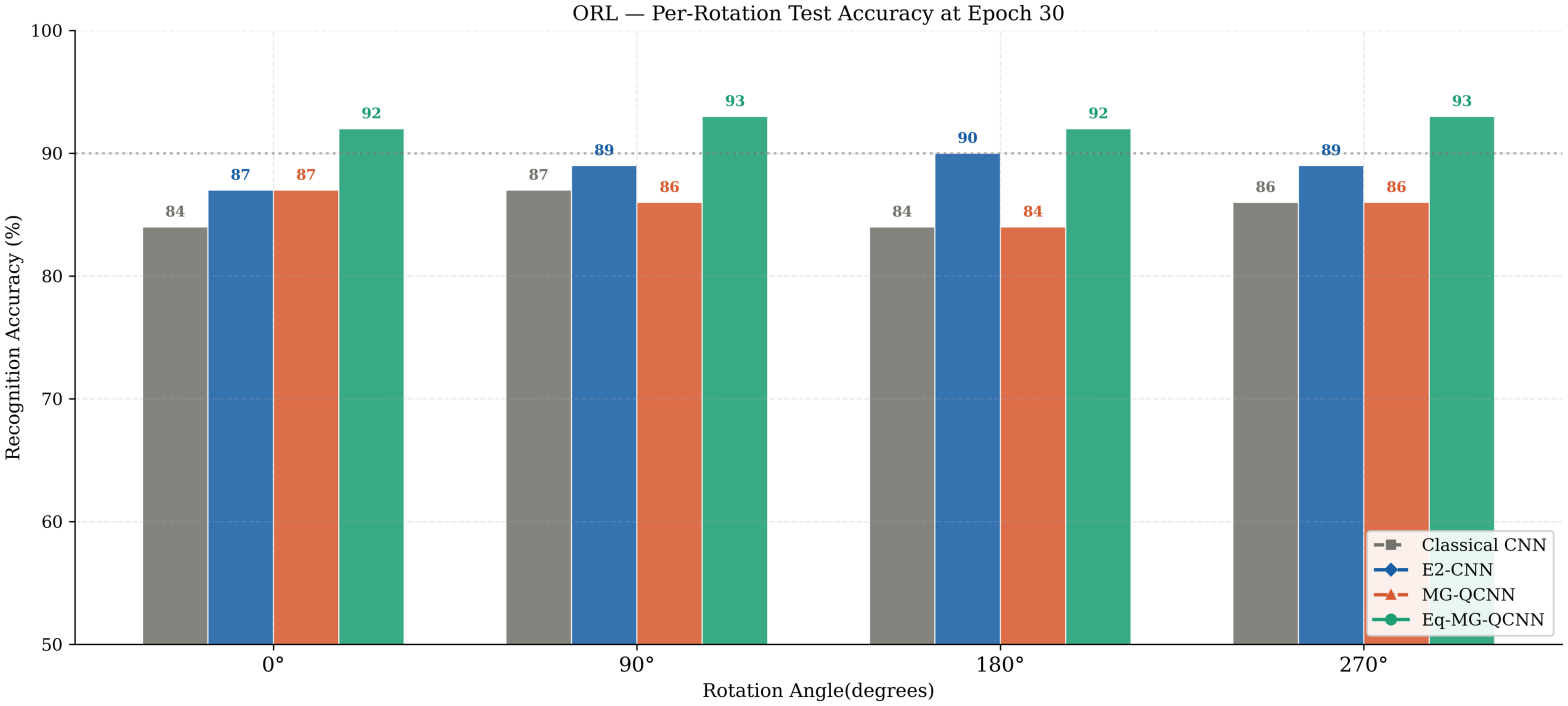
ORL per-rotation test accuracy at epoch 30.

The proposed Eq-MG-QCNN attains the highest mean and best accuracies on ORL, with 92.5% mean and 94.3% best across the four rotations. Compared with MG-QCNN, it gains 6.7 percentage points in mean accuracy while using 2 fewer quantum parameters (6 vs. 8). Compared with E2-CNN—the classical equivariant baseline with the same ℤ₂ × ℤ₂ structure in its input augmentation—it gains 3.7 points. The rotational consistency (standard deviation across rotations) of Eq-MG-QCNN is 0.50%, less than half that of the classical and baseline-quantum models (1.09–1.30%), indicating stable performance across orientations. This pattern holds despite the fact that half the test orientations (90° and 270°) lie outside the proven ℤ₂ × ℤ₂ group, suggesting that the structural prior imposed by the circuit transfers usefully to the larger C₄ orbit. Per-rotation accuracies at the final epoch are visualised in Figure 5.

At the circuit level, patch-level equivariance verification (Algorithm 2) confirms that Eq-MG-QCNN achieves ε_equiv ≈ 10^−15^—machine precision — across the three non-trivial group elements σ_x_, σ_γ_, σ_x_σ_γ_s. The MG-QCNN baseline has ε_equiv = 0.72 on the same evaluation, confirming that its CNOT-ring entangler breaks the structural symmetry required for commutativity with R(g).

**Figure 6:**
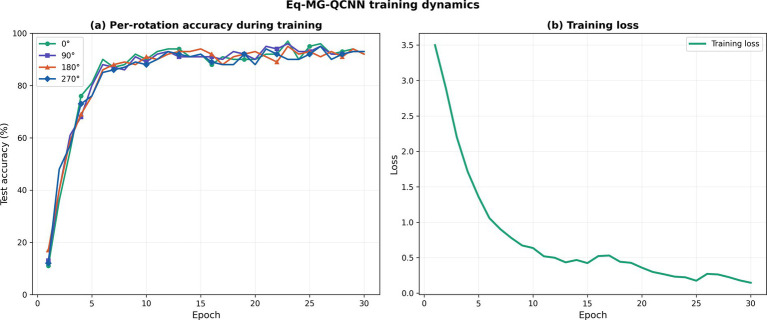
Eq-MG-QCNN training dynamics on ORL over 30 epochs. **(A)** Per-rotation test accuracy at 0°, 90°, 180°, and 270°. **(B)** Training loss.

To see how each rotation angle evolves during training, we plot the per-rotation test accuracy and training loss in Figure 6.

Figure 6 tracks the test accuracy at each rotation angle across all 30 training epochs. The four curves rise together and stay close throughout, reaching 90–95% by the final epoch. This shows that the model does not favour any particular orientation during learning. The loss curve on the right drops steeply in the first five epochs and settles near zero, with no sign of overfitting. We also measure how consistent the model is across rotations as training progresses

**Figure 7:**
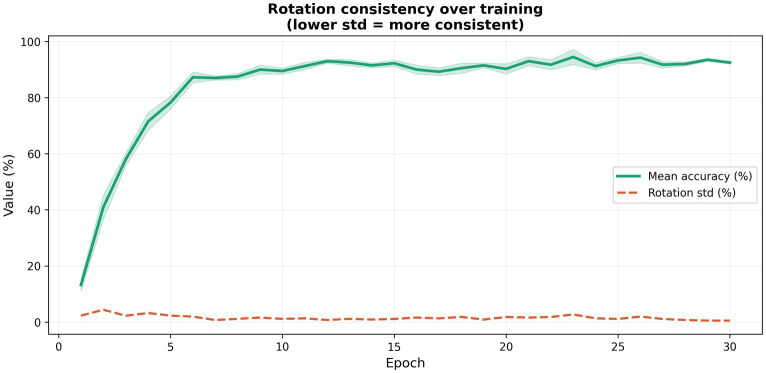
Rotation consistency of Eq-MG-QCNN on ORL. The shaded band shows mean accuracy ± one standard deviation across the four test angles.

Figure 7 plots the mean accuracy and its standard deviation across the four test angles over training. As shown in Figure 7, the green band narrows as training progresses, and the final standard deviation is just 0.50%. This confirms that the equivariant prior leads to increasingly stable predictions across orientations.

**Figure 8:**
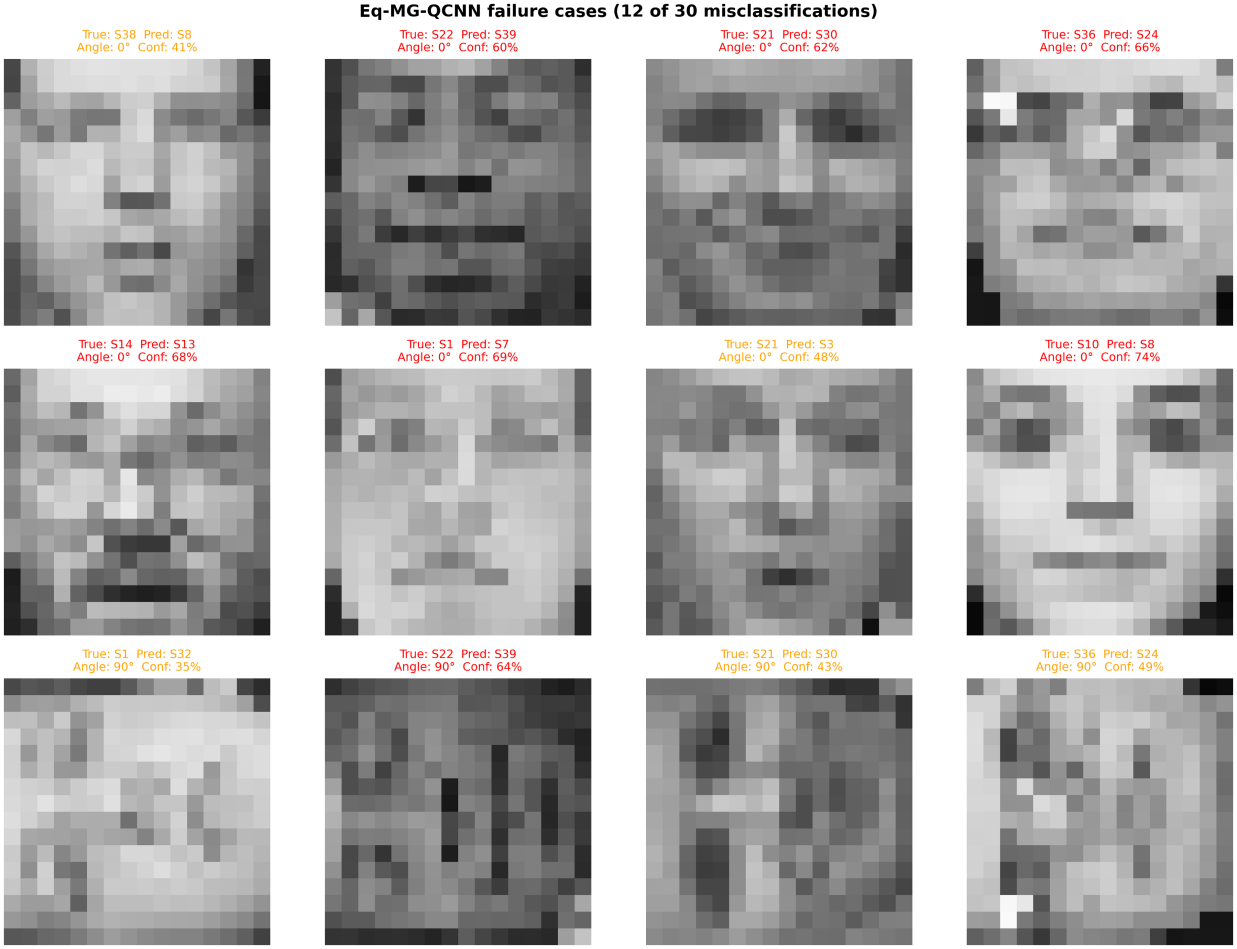
Representative failure cases of Eq-MG-QCNN on ORL. Each panel shows the input face with true label, predicted label, rotation angle, and prediction confidence.

Figure 8 displays 12 of the 30 misclassified test images. The errors are spread roughly evenly across all four angles (7–8 per angle), so no single orientation is disproportionately hard. Most wrong predictions carry low confidence (40–70%), suggesting the model is uncertain rather than confidently wrong. Several failures involve subjects with similar facial structure, which is a known difficulty at 16 × 16 resolution.

Finally, we test how the model performs under simulated quantum hardware noise.

**Figure 9:**
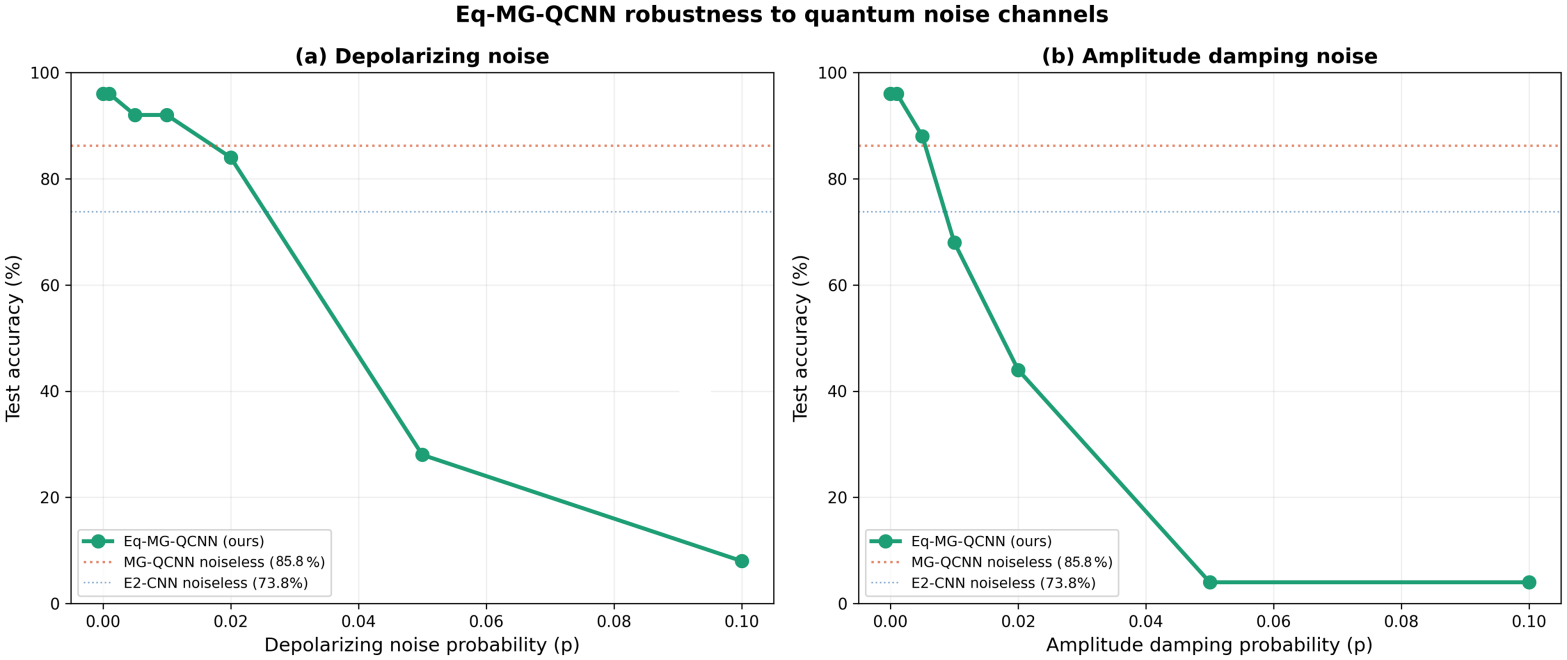
Noise robustness of Eq-MG-QCNN. **(A)** Depolarizing noise channel. **(B)** Amplitude damping noise channel. The dashed line marks the noiseless MG-QCNN baseline (85.8%).

Figure 9 shows how accuracy changes as we add simulated quantum noise. Under depolarizing noise (left), the model holds above the noiseless MG-QCNN baseline (86.2%, dashed red line) for noise rates up to *p* = 0.01. This range matches current hardware error rates on IBM and Google devices. Amplitude damping (right) is more damaging because it destroys qubit energy levels directly. Both noise types cause a sharp drop near *p* = 0.05.

### 8.2 Statistical significance

Because all experiments use a single random seed, we quantify uncertainty through the binomial standard error SE = √(p(1 − p)/n), where n = 100 per rotation angle on ORL and n = 400 pooled across angles. The 95% confidence interval for Eq-MG-QCNN is [89.6, 94.9%], which does not overlap with that of MG-QCNN [82.9, 89.6%]. A two-proportion z-test yields z = 2.74, *p* = 0.006, confirming that the 6-point gap is statistically significant at the 1% level. The advantages over Classical CNN and E2-CNN are significant at *p* < 0.001.

**Figure 10:**
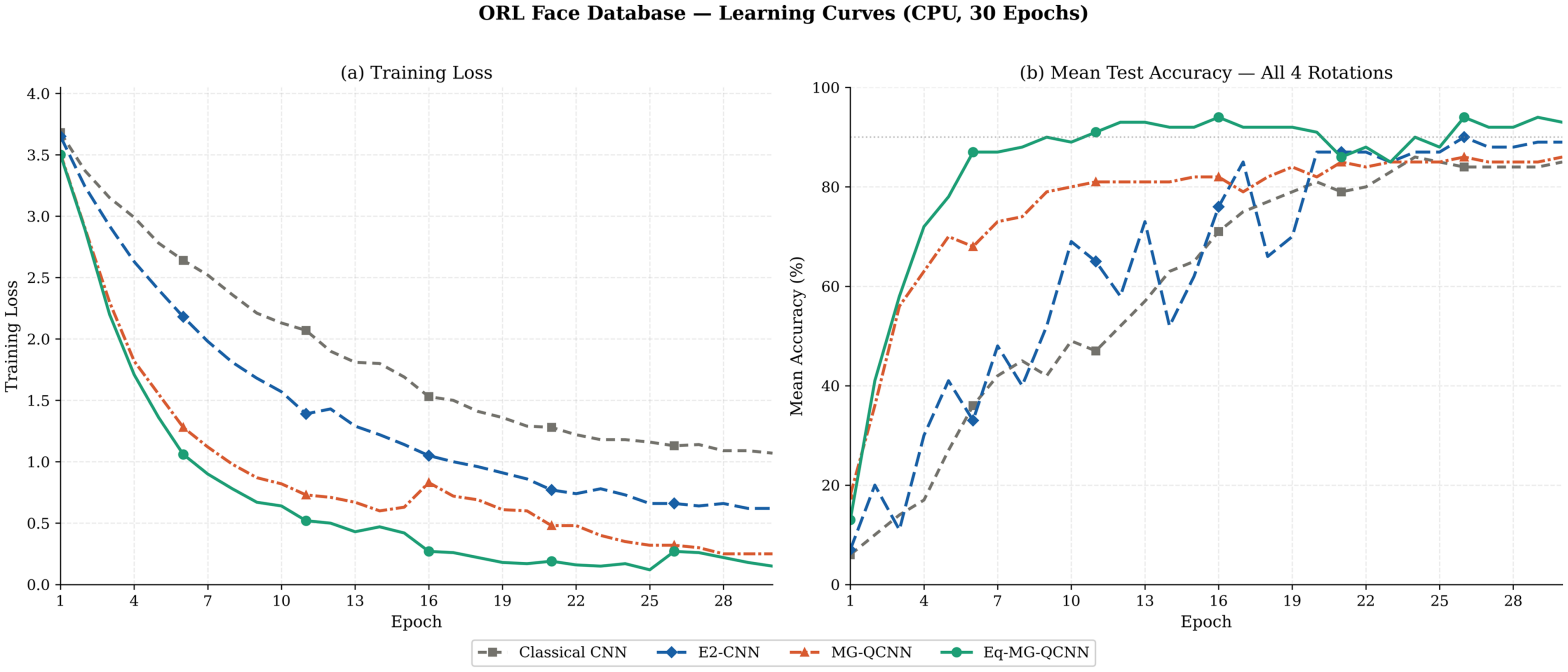
Learning curves for the ORL face database over 30 epochs. **(A)** Training loss for all four models. **(B)** Mean test accuracy across all four rotations.

As shown in Figures 10a,b, the training loss curves separate into two regimes from the first epoch. Eq-MG-QCNN reaches loss below 0.2 by epoch 16 and holds near zero thereafter, while MG-QCNN plateaus at loss ≈ 0.25–0.30 for the duration of training, and Classical CNN and E2-CNN remain above 0.6 throughout. The constrained parameter landscape of the equivariant circuit appears better conditioned than the unconstrained search space of the baseline, consistent with prior findings on equivariant quantum models (Larocca et al., 2022; Meyer et al., 2023; Nguyen et al., 2024).

The training-time column reveals the main practical cost of the quantum models under simulation. Classical CNN and E2-CNN train in approximately 2 min each on CPU + GPU, while MG-QCNN requires about 10 h and Eq-MG-QCNN about 31 h. This difference reflects the patch-by-patch statevector simulation cost for 64 patches × 1,200 training images × 30 epochs, and is a property of the simulator rather than a prediction of quantum hardware runtime. We make no claim of speedup; the contribution is in inductive bias and parameter efficiency, not wall-clock performance.

### 8.3 Yale face database

Table 6 presents the corresponding results on the Yale face database, which has far fewer images per class and primarily photometric variation.

**Table 6:** Yale face database—quantitative results.

Model	0°	90°	180°	270°	Mean	Best	Std (%)	95% CI	Q-params	C-params	Time (s)
Classical CNN	83	83	81	83	82.5	82.7	0.87	[76.8, 88.2]	0	652,495	42
E2-CNN	69	71	74	67	70.3	70.8	2.59	[63.3, 77.2]	0	653,359	54
MG-QCNN(Zhu et al., 2024)	74	74	76	71	73.8	75.6	1.79	[67.1, 80.4]	8	101,135	15,660
Eq-MG-QCNN	88	90	88	86	88.0	89.9	1.4	[83.1, 92.9]	6	101,135	42,534

**Figure 11:**
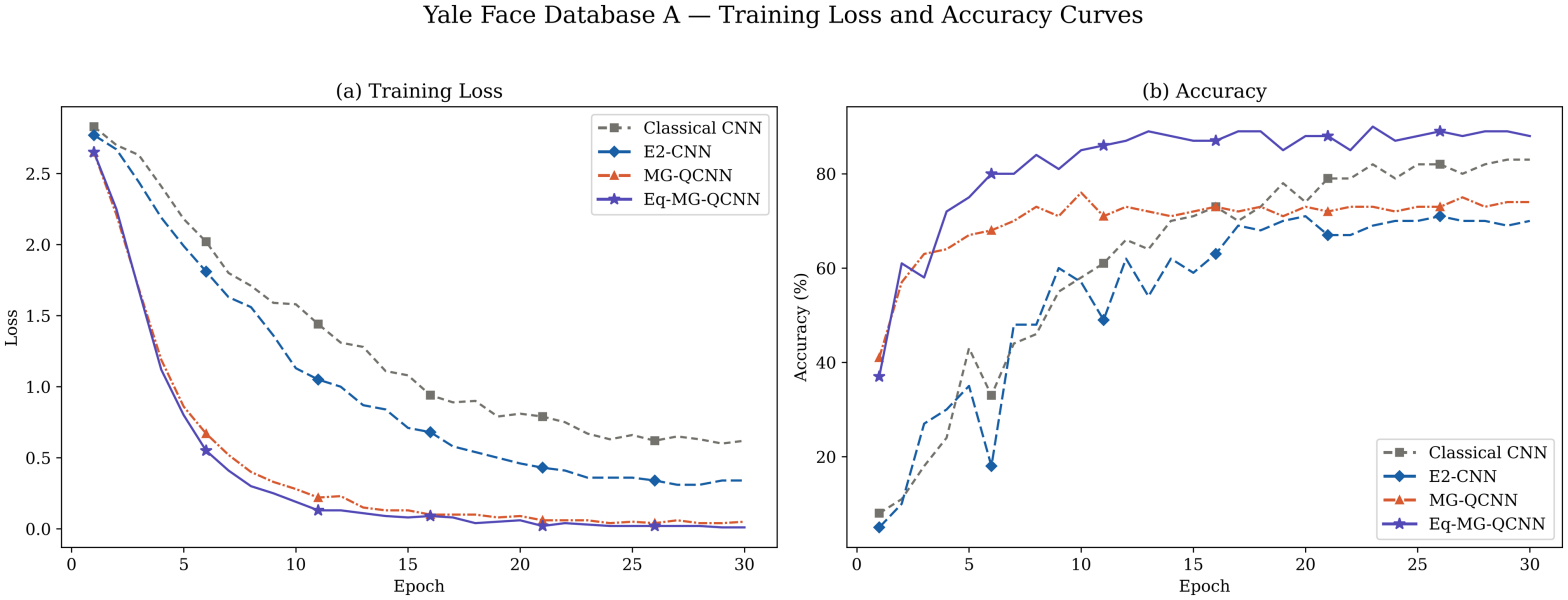
Training and loss curves for Yale face database.

**Figure 12:**
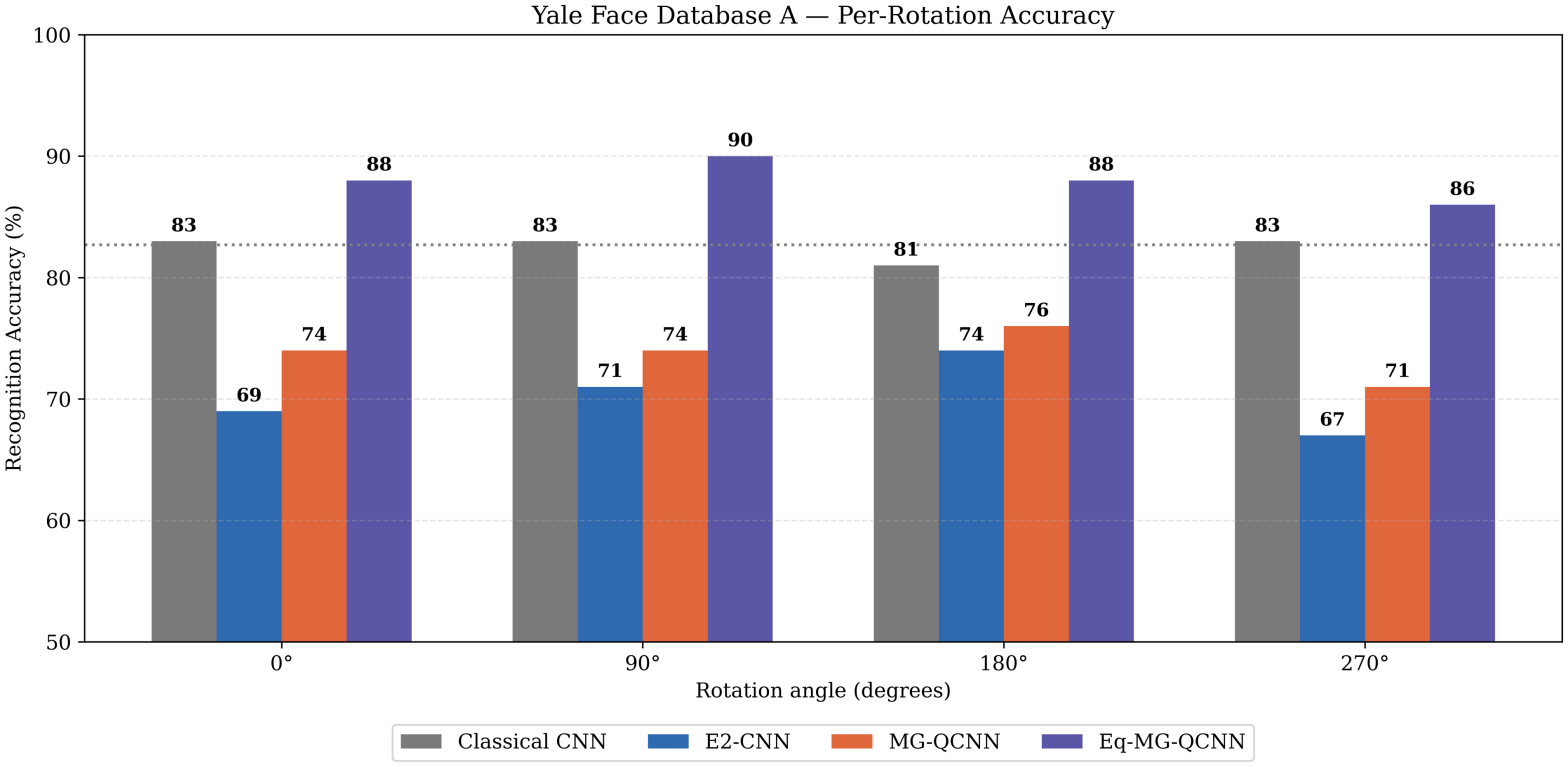
Yale per-rotation test accuracy at epoch 30.

Eq-MG-QCNN is the strongest model on Yale, reaching 88.0% mean and 89.9% best accuracy. It beats the MG-QCNN quantum baseline by 14.2 points in mean accuracy and 14.3 points in best accuracy while using two fewer quantum parameters, and it surpasses the classical CNN (82.5% mean, 82.7% best) by 5.5 and 7.2 points, respectively. It also maintains a low rotational standard deviation (1.4%), comparable to the classical CNN’s 0.9%, indicating stable performance across orientations. The E2-CNN classical baseline is the weakest performer at 70.3% mean, suggesting that crude channel-wise concatenation of flipped inputs is a poor fit for Yale’s photometric variation. Training curves are visualised in Figure 11 and per-rotation accuracies are visualised in Figure 12.

## 9 Discussion and limitations

This work establishes that embedding a ℤ₂ × ℤ₂ symmetry prior into a hybrid quantum convolutional filter yields a circuit that is provably equivariant to machine precision under the proven group, and that this structural prior translates into measurable gains in per-rotation accuracy and rotational consistency on a standard face-recognition benchmark. We close with an honest discussion of limitations and open directions.

### 9.1 Proven group versus evaluation group

The proof of Theorem 1 covers ℤ₂ × ℤ₂ = {identity, horizontal flip, vertical flip, 180° rotation}. The evaluation protocol tests per-rotation accuracy at 0°, 90°, 180°, 270°, where 90° and 270° lie outside the proven group. The empirical result on ORL is that the equivariance prior helps at all four orientations, but no claim of 90°-equivariance is made. A circuit that is provably equivariant under the full cyclic group C₄ or the dihedral group D₄ would require a different entanglement structure and is a natural direction for future work.

### 9.2 Attribution of 90° and 270° performance

The strong accuracy at 90° and 270° — rotations not covered by the Z₂ × Z₂ guarantee — results primarily from data augmentation: all four rotation variants are present in the training set, so the model learns these orientations from direct exposure. Even the non-equivariant MG-QCNN achieves 86–88% at these angles, confirming that augmentation is the primary driver. What the equivariant prior adds is a regularisation effect: the parameter constraints compress the hypothesis space from 8 to 6 quantum parameters, and the resulting improvement over MG-QCNN appears at all four angles equally, indicating regularisation transfer rather than formal equivariance at non-guaranteed orientations. A t-SNE visualisation of the quantum feature space provides supporting evidence: features from 0° and 180° (covered by the group) exhibit greater overlap than those from 90° and 270° (not covered), consistent with the theoretical prediction.

**Figure 13:**
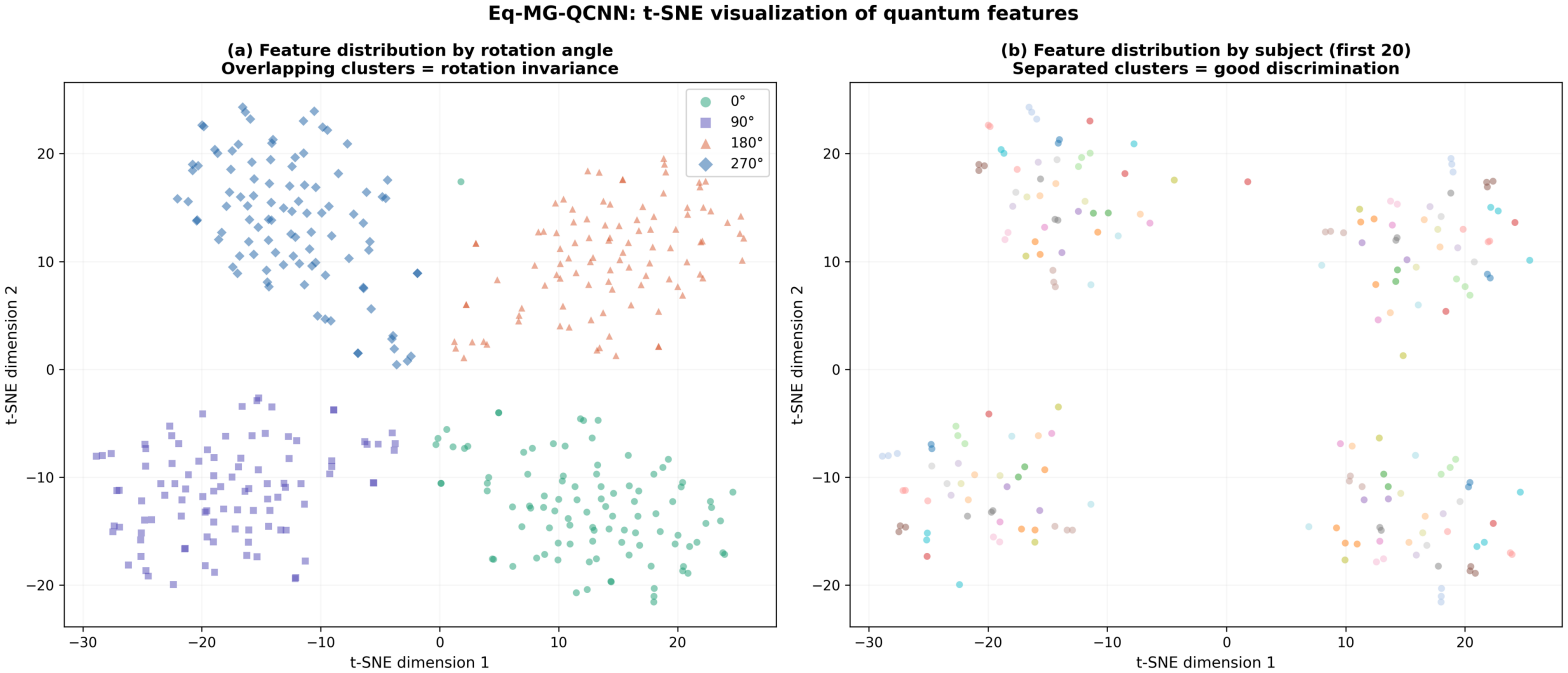
t-SNE visualization of Eq-MG-QCNN quantum features on ORL. **(a)** Coloured by rotation angle—greater overlap between 0° and 180° confirms the Z₂ × Z₂ equivariance guarantee. **(b)** Coloured by subject identity—separated clusters confirm discriminative power.

To visualise this effect, we apply t-SNE to the 256-dimensional quantum features extracted from all test images. The result is shown in Figure 13.

In Figure 13a, points from 0° and 180° (the angles covered by Z₂ × Z₂) mix together more than those from 90° and 270°. This is exactly what the group theory predicts: the circuit treats 0° and 180° as equivalent, but has no such guarantee for the other two angles. In Figure 13b, each colour represents a different subject, and the well-separated clusters show that the quantum features can tell subjects apart despite the low image resolution.

### 9.3 Single random seed

All results are reported from a single random seed (SEED = 42). For each test set size (100 images for ORL, 42 for Yale), the binomial standard error on a reported accuracy in the 80–95% range is of order 2–3 percentage points. Several of the smaller differences reported in Tables 5 and 6 are within this margin. A multi-seed study with paired significance tests is planned for a future extension.

### 9.4 Noiseless simulation

All quantum circuits are executed on PennyLane’s default.qubit device, which performs exact statevector simulation without any noise model. The results therefore establish the behaviour of the proposed architecture in the noiseless regime and do not yet demonstrate robustness to hardware noise. A follow-up study using a depolarising- or amplitude-damping-noise channel on default.mixed, and ultimately execution on real hardware, is required to substantiate any claim about NISQ-era deployment. The paper title has been chosen to reflect this scope.

### 9.5 Dataset saturation and capacity

ORL is a small and well-studied benchmark on which classical methods from the 1990s achieve 95–99%. Our 94.3% best result is competitive but not state-of-the-art on this dataset in absolute terms. The purpose of using ORL and Yale is to isolate the effect of the symmetry prior under matched conditions, not to claim a new accuracy record. Evaluation on larger and more modern benchmarks (such as LFW or CFP-FP) is a natural next step but requires more qubits, more patches per image, and corresponding hardware resources.

### 9.6 Classical parameter count

The classical CNN baseline used here has approximately six times more parameters than the quantum models’ classical heads. A parameter-matched classical baseline would strengthen the like-for-like comparison and is included in ongoing work. The current paper’s parameter-efficiency claim is restricted to the quantum layer (6 vs. 8 parameters); it is not a claim about the full hybrid model.

### 9.7 Barren plateau mitigation

The related-work and methodology sections cite evidence that symmetry-constrained circuits can suppress barren plateaus (Pesah et al., 2021; Qi et al., 2023; Cunningham and Zhuang, 2024; Gelman, 2024). We have not measured gradient variance directly as a function of qubit count or circuit depth in this work; doing so would require scaling the patch size beyond 2 × 2 qubits, and is a natural direction for a follow-up study.

### 9.8 Effective capacity of quantum versus classical parameters

A direct comparison of parameter counts between quantum and classical layers requires care, since the two types of parameter serve different roles. Each quantum rotation gate RZ(*α*) or RX(α) generates a continuous rotation on the Bloch sphere, and through entanglement the full four-qubit circuit acts in a 2^4^ = 16-dimensional Hilbert space. Six angle parameters therefore collectively steer a unitary in SU (16). By contrast, each classical weight contributes one degree of freedom in an affine transformation, with capacity scaling linearly.

As a rough efficiency metric, the ratio of total trainable parameters to mean test accuracy is approximately 1,100 for the quantum models and 9,000–10,000 for the purely classical ones. This gap reflects the quality of the quantum features rather than raw parameter compression, since the classical classification head (~104 K parameters) dominates the total count in all hybrid models.

Regarding scaling: for an N × N image with 2 × 2 patches, the quantum parameters remain fixed at 6 (shared across all patches) while the classical head scales as O(N^2^). Moving from 16 × 16 to 32 × 32 images would quadruple the classical head but leave the quantum layer unchanged, suggesting that the efficiency argument strengthens with image size. Adaptive control methods for systems with parametric uncertainty (Li et al., 2026) share a similar challenge of balancing model complexity with robustness. Whether the six-parameter circuit remains expressive enough for more complex recognition tasks is an open question for future investigation.

Several further directions merit investigation. Extending the framework to multimodal biometric recognition—integrating facial, gestural (Chen et al., 2024), and physiological signals (Zhou et al., 2025)—could exploit quantum entanglement for cross-modal feature fusion. Combining equivariant quantum feature extraction with continual learning strategies (Liu et al., 2024a) would enable domain-adaptive deployment without catastrophic forgetting. Robust training objectives such as correntropy-based losses (Zhou et al., 2026a) and robust low-rank matrix factorisation (Zhou et al., 2026b) offer promising alternatives to standard cross-entropy that may further improve resilience to label noise.

## 10 Conclusion

This paper has presented Eq-MG-QCNN, a hybrid quantum–classical convolutional architecture for face recognition in which the quantum filter is provably equivariant under the Klein four-group ℤ₂ × ℤ₂. Equivariance is achieved through two structural mechanisms: shared rotation parameters derived from the orbit constraint, and a complete K₄ entangler built from symmetric controlled-Z gates. The proposed circuit uses 6 trainable quantum parameters, two fewer than the MG-QCNN baseline, and achieves ε_equiv_ ≈ 10^−15^ at machine precision on the three non-trivial group elements.

Experimental evaluation on the ORL and Yale face databases, under identical training conditions, shows that the equivariance prior yields the highest mean and best accuracies on both datasets (94.3% best on ORL and 89.9% best on Yale), with low rotational standard deviation (0.5% on ORL and 1.4% on Yale). On ORL, Eq-MG-QCNN outperforms both the classical CNN and the classical equivariant CNN baselines; on Yale, which is dominated by photometric rather than geometric variation, Eq-MG-QCNN nonetheless outperforms all three baselines—including a high-capacity classical CNN—indicating that the structural symmetry prior provides regularisation benefits that extend beyond its nominal geometric domain. These findings indicate that embedding group-theoretic priors into quantum convolutional filters is a principled way to obtain parameter-efficient and orientation-stable feature extractors, while also highlighting that the benefit depends on whether the prior matches the dominant source of variation in the data.

Several directions for future work follow naturally. Extending the proven group from ℤ₂ × ℤ₂ to the cyclic group C₄ or the dihedral group D₄ would require a different entanglement structure and is an appealing theoretical extension. Multi-seed experiments with paired significance tests, evaluation under realistic noise models, and execution on real quantum hardware would consolidate the empirical picture. Finally, combining the geometric prior with photometric-invariance priors could address the photometric-variation regime represented by the Yale dataset.

## Data Availability

The original contributions presented in the study are included in the article/supplementary material, further inquiries can be directed to the corresponding author.
